# Investigation of the Renal Defensive Influence of Walnut Septa Extract Against Acute Renal Ischemia/Reperfusion Injury

**DOI:** 10.1155/mi/9713697

**Published:** 2025-06-12

**Authors:** Seda Askin, Khasiiat Iminova, Bahri Avci, Hakan Askin

**Affiliations:** ^1^Health Services Vocational School, Ataturk University, Erzurum, Türkiye; ^2^Department of Molecular Biology and Genetics, Ataturk University, Erzurum, Türkiye

**Keywords:** acute renal failure, ischemia/reperfusion, renoprotective, walnut seota extract

## Abstract

**Aim:** Acute kidney injury (AKI), also known as renal failure among the public, is the sudden decrease or loss of kidney functions. In the treatment of this condition, drugs with anti-inflammatory properties are generally preferred, but these drugs have many side effects. Physicians may need natural antioxidants as an alternative or additional treatment to the applied treatment to eliminate or reduce these side effects. Therefore, we conducted our current study molecularly to reveal the protective potential of walnut septa extract (WSE) in targeting oxidative stress, inflammation, ferroptosis, and cellular differentiation mechanisms in kidney tissues in Sprague-Dawley rats with kidney damage.

**Methods:** The bioactive compounds in this extract were identified. Before inducing a renal IR injury model in the rats this extract was administered. Then, tissue oxidative stress markers were detected by ELISA. Following the treatment, molecular analyses were performed to determine antioxidant, anti-inflammatory, ferroptosis, and cellular differentiation activities in kidney tissues. Gene expression levels of kidney injury molecule-1 (*Kim-1*), nuclear factor erythroid 2 (*Nrf2*), lipocalin 2 (*Lcn2*), glutathione (GSH) peroxidase 4 (*Gpx4*), and keratin 8 (*Krt8*) were assessed.

**Results:** The primary bioactive compound identified in this extract was β-sitosterol, accounting for 62.066% of the total extract. Pretreatment with WSE led to an increase in GSH activity and a reduction in lactate dehydrogenase (LDH) levels in the kidney tissues. On a molecular level, this extract promoted the activation of *Gpx4*, *Krt8*, and *Nrf2* genes, while inhibiting the expression of *Kim-1* and *Lcn2* genes, indicating its protective effects. Extract was shown to exert renoprotective effects by reducing oxidative stress (*Gpx4* and *Nrf2*), suppressing inflammation (*Lcn2* and *Kim-1*), and supporting cellular structure and apoptosis regulation (*Krt8*).

**Conclusion:** These findings suggest that extract could be a promising therapeutic candidate for renal ischemia-reperfusion (RIR) injury. Further comprehensive and long-term studies are recommended to validate these findings.

## 1. Introduction

Acute kidney injury (AKI) due to ischemia-reperfusion injury is a serious pathological process that occurs when blood flow to the kidneys is reduced (ischemia) and then, blood flow is restored (reperfusion). This process can cause significant damage to renal tissues and lead to AKI. The ischemic phase causes oxygen and nutrient depletion, causing cellular damage and dysfunction, while the sudden influx of oxygen during reperfusion can cause further tissue damage by increasing reactive oxygen species (ROS). This, combined with inflammatory responses and endothelial dysfunction, leads to cellular apoptosis and necrosis. The combination of these processes creates a complex pathophysiological condition that contributes to the progression of AKI [1, 2]. Renal ischemia-reperfusion (RIR) injury is associated with high morbidity and mortality rates, but effective treatments are still lacking [3]. RIR may contribute to the progression of renal dysfunction, leading to the development of AKI in native kidney tissue and renal allograft transplants. Reperfusion of the ischemic kidney results in increased oxidative stress and aggravated inflammation, damaging cellular DNA, protein, and cellular integrity, resulting in necrosis or apoptosis. Therefore, the use of antioxidant compounds may be a promising approach to alleviate RIR-induced oxidative stress [4]. Herbal products may have positive effects not only against cellular damage caused by oxidative stress but also on the immune system due to their immunomodulatory effects. This suggests that they may be a potential treatment tool, especially against damage to organs such as the kidneys. Recent studies suggest that products derived from plants may have a protective effect against RIR damage, thus, improving kidney function and minimizing tissue damage [5]. Herbal medicines are increasingly used by people due to their good properties such as having few side effects and not causing adverse reactions. For this reason, the production and use of herbal medicines should be based on scientific data and include the most up-to-date biological screening techniques along with analytical methods [6]. Walnuts are members of the Juglandaceae family and have high mineral, protein, fat, and vitamin content. They also contain mainly polyphenols, flavonoids, and sterols [7 ]. Walnut septa (WS) is also called seed coat, seed skin, or walnut clip. This part has the highest phenolic content among other parts of the walnut, including the edible part. It is located inside the walnut kernel and has a leathery structure that contains approximately 5% of a walnut. It has a brown tone, curved, scaly appearance, slightly brittle, and felt-like appearance. Previous in vitro and in vivo studies have suggested that this WS part has antimicrobial, hypolipidemic, hypoglycemic, antioxidant, antitumor, immune system strengthening, hematopoiesis stimulating, and antiaging effects [8–10]. In previous tests and trials using this extract on rats, no acute or subchronic toxicity was observed [11]. In our previous study, it was clearly shown that walnut fruit diaphragm extract has a protective effect in hyperlipidemia-induced male Wistar rats [6]. While the full mechanism of action is still under investigation, WSE seems to exert its effects through a combination of antimicrobial, antioxidant, anticancer, anti-inflammatory, and neuroprotective activities. Further research, especially clinical trials, is needed to confirm these mechanisms and explore potential therapeutic applications more thoroughly. At the molecular level, WS extract (WSE) likely alleviates oxidative stress and inflammation by scavenging free radicals, modulating inflammatory signaling pathways, enhancing antioxidant defenses, and protecting cellular structures. These mechanisms combine to reduce oxidative damage and inflammation, which are linked to a variety of chronic conditions, including cardiovascular diseases, diabetes, and neurodegenerative disorders [12]. Considering all these factors, we aimed to investigate whether the ethanolic extract of WS (E-WSE) exhibited a protective effect against ischemia/reperfusion-induced damage in rat kidney tissues.

## 2. Material and Methods

### 2.1. Preparation and Storage Procedure of WSE

WSE, obtained from walnuts collected from Tortum district of Erzurum province located in the northeast of Turkey, was the main material of the presented study. First, the outer shells of the walnuts were removed and the samples were dried as a thin layer at room temperature without being exposed to direct sunlight. To obtain the extract, the endocarp membranes were ground into fine powder and extracted with ethanol. Approximately 62.762 g of extract was obtained from a total of 384 g of WS and the extraction efficiency was calculated as 16.344%. To increase extraction efficiency and reduce yield variability, many variables (solvent choice, time, particle size, raw material variability, process conditions, environmental factors, etc.) were controlled and the extraction process was optimized. The obtained extract was stored at +4°C. Fifty grams of walnut seed shell was dissolved in 1000 mL of ethanol and stirred for 3 days on a magnetic stirrer. Then, it was extracted for 4 h between 60 and 80°C. After the process, the solid material was separated with a sieve and the solvent was evaporated at 155 rpm at 50°C (Heidolph, Schwabach, Germany). The obtained extract was dried in an oven (Binder, Tuttlingen, Germany) and stored at +4°C.

### 2.2. Analysis of Phytochemical Components of WSE

Gas chromatography–mass spectrometry (GC–MS) analysis was performed to identify the phytochemical content. A GC system combined with a mass selective detector and a GC autosampler was used for all analyses. Briefly, 1 µL of extract was injected into a GC column equipped with a capillary column (HP-5 MS, 0.25 µm, 30 m × 0.25 mm). The injection temperature and detector temperature were set at 250°C. Helium was used as the carrier gas at a constant flow rate of 1 mL/min. The oven temperature was programmed as 50°C for 1 min, 100°C at 20°C per minute for 1 min, 180°C at 10°C per minute for 1 min, 220°C at 5°C per minute for 5 min, and 300°C at 10°C per minute for 5.5 min. Mass spectra were recorded using a mass spectrometer in the 50–600 MHz scanning range [13].

### 2.3. Animals and Surgical Procedure

In the study, 18 female Sprague-Dawley rats (weight range 250–350 g) obtained from the Experimental Animals Unit of our university were used. The rats participating in the experiment were kept in groups of six in polycarbonate cages with room temperature (22 ± 2°C) and a 12-h light–dark cycle, with free access to water and food until the beginning of the experiment. The care and use of animals in experiments are regulated in accordance with national guidelines. Every effort was made to minimize the suffering of the animals during the experiment. Rats were divided into three experimental groups (A–C) according to their weight (*n* = 6 in each group). (A) Healthy control (HC): Animals in this group were not subjected to any intervention and were observed under completely natural conditions. (B) Renal IR: Experimental ischemia-reperfusion induction was applied to this group. (C) Renal IR + WSE: Before ischemia-reperfusion application to animals in this group, WSE was administered via gavage at a dose of 450 mg/kg/p.o. The dose of the extract was determined based on previous studies and recommendations in the literature [8]. While WSE was administered orally to the treatment group 1 h before IR procedure, only solvent (1% carboxymethyl cellulose solution) was administered to the HC group. After the rats were anesthetized via intraperitoneal injection of a combination of ketamine (75 mg/kg, i.p.) and xylazine (10 mg/kg, i.p.) [14], the operation area was shaved and prepared for surgery. Clips were placed to stop blood flow in the kidneys and kept for 50 min. After this period, the clips were removed and the kidneys were left to reperfuse for 3 h. All procedures except clamp placement were applied in the same manner to the animals in the HC group [14].

The rats were then slaughtered and the kidney tissues were removed. The removed tissues were cleaned by washing with saline, then, dried and treated with liquid nitrogen and stored at 80°C until further molecular assessment. 0.2 g of kidney tissue from animals belonging to separate groups was weighed and placed in Eppendorf tubes. Homogenate beads and phosphate buffer (pH = 7.2) were added. It was homogenized with four cycles (1 min shaking, 10 s waiting) at 4000 rpm in the BeadBlaster device. Then, it was centrifuged at 13,000 rpm, +4°C, for 30 min. In addition, the homogenization buffer was 1 mL in volume and contained 0.1 M KH_2_PO_4_ and 10 mM EDTA. The supernatant obtained after centrifugation was divided into two equal groups for further processing and stored at −80°C, thus, preserving the structural integrity and biochemical properties of the tissue. This process provided the most suitable conditions for further analysis [15].

### 2.4. Experiments on Oxidative Stress Using ELISA

The levels of glutathione (GSH, cat. no. A005-1-2) and lactate dehydrogenase (LDH, cat. no. A020-2-2), both sourced from Nanjing Jiancheng Bioengineering Institute, were measured using the respective kits following the manufacturer's protocols. All procedures were performed on homogenized kidney tissues [16].

### 2.5. RNA Isolation From Kidney Tissue

RNA purification was performed using the EcoPURE RNA Isolation Kit provided by EcoTech. Approximately 0.03 g of kidney tissue was used in this process. The tissue was first cut into large pieces and then placed in RNase-free Eppendorf tubes. Four hundred microliters of lysis buffer, β-mercaptoethanol and homogenization beads were added to the tubes. Homogenization was performed by mixing the tubes at 3000 rpm in a homogenizer for five cycles. After homogenization, the tubes were removed from the device and centrifuged at +4°C at 13,000 rpm for 25 min. The supernatant formed after centrifugation was carefully transferred to RNase-free Eppendorf tubes and separated from the pellet part. An equal volume of ethanol was added to the supernatant and vortexed for 10 s. Seven hundred microliters of this mixture was placed in a column and centrifuged for 2 min at maximum speed. The RNA retained in the column was isolated and the remaining liquid was removed. Four hundred microliters of wash buffer-1 and 500 µL of wash buffer-2 were added to the column and each wash was centrifuged for 30 s at maximum speed. As the final wash step, 200 µL of wash buffer-2 was added to the column and centrifuged for 2 min. Finally, 50 µL of elution buffer was added directly to the center of the column to obtain the RNA on the column. After this step, the column was left at room temperature for 1 min and then, centrifuged for 30 s at maximum speed to obtain RNA. Nanodrop device was used to determine the concentration and purity values of the obtained RNAs. Before the measurement, 2 μL of elution buffer (as a blank control) was dropped onto the device plate and then 2 μL of RNA isolated from tissues for each sample was placed on the plate and measurements were performed. Purified RNA samples were stored at −20°C for immediate use or −80°C for long-term storage [17].

### 2.6. cDNA Extraction

iScript cDNA Synthesis Kit (Bio-Rad) was used for cDNA synthesis. For the process, 4 µL of 5x iScript reaction mixture was placed in a tube. 1 µL iScript reverse transcriptase enzyme, 3 µL RNA template, and 12 µL nuclease-free water were added to it, respectively, and the total volume was adjusted to 20 µL. The prepared PCR tubes were placed in the SensoQuest Thermal Cycler and the PCR stage, which included the initiation stage, reverse transcription, and enzyme inactivation steps, was performed. After these steps were completed, cDNA synthesis was successfully performed. The purity and concentration of the obtained RNAs were measured with the Nanodrop device [18].

### 2.7. Gene Primer Design and Real-Time PCR

In our study, we used kidney injury molecule-1 (*Kim-1*), nuclear factor erythroid 2 (*Nrf2*), lipocalin 2 (*Lcn2*), GSH peroxidase 4 (*Gpx4*), and keratin 8 (*Krt8*) genes, as well as *Gapdh* as a housekeeping gene. Before the real-time PCR process, lyophilized gene primers were prepared for the process as written in the protocol. The primers of the genes used are shown in Table 1.

Real-time PCR analysis was performed using the Bio-Rad kit. The process was completed by performing three replicates for each tube. For each sample, 18 µL of SYBR Green Master Mix was transferred to PCR tubes and 2 µL of sample CDNA was added. These samples prepared for the real-time PCR process were placed in the Rotor-Gene PCR device and the cycles were set.

### 2.8. Performing Statistical Analyses

All measurements were performed in triplicate for each tissue and sample. For relative quantification of gene expression we used the comparative *ΔΔ*Ct method of Livak and Schmittgen [19]. Gapdh expression was used for normalization and relative quantification. Comparison of results was performed by one-way ANOVA and Tukey's honestly significant difference (HSD) test with Prism software (GraphPad 8.0.2 Software, San Diego, CA). Statistically significant differences are presented as follows: ns *p*  > 0.05 (not significant); *⁣*^*∗*^*p*  < 0.05 (significant); *⁣*^*∗∗*^*p*  < 0.01 (very significant); *⁣*^*∗∗∗*^ or ^*∗∗∗∗*^*p*  < 0.001 or 0.0001 (extremely significant) [20].

## 3. Results

### 3.1. GC–MS Analysis Findings

**Figure 1:**
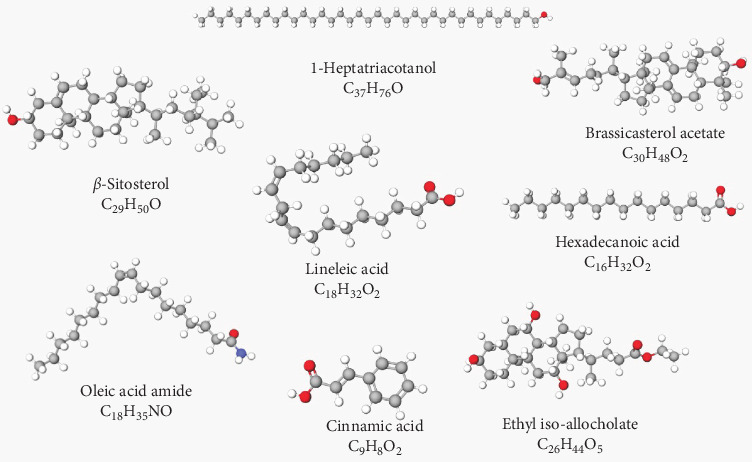
Structures of phytocompounds determined by GC–MS analysis.

In the GC–MS analysis performed on the WS ethanol extract, the presence of various bioactive substances such as alcohols, mono- and polyunsaturated fatty acids, esters, phytosterols, and phenolic compounds was detected (Table 2). The structures of the bioactive compounds determined by GC–MS analysis are shown in Figure 1.

Among these compounds, β-sitosterol (62.066%) was found to be the compound with the highest rate, while linoleic acid (0.564%) was found to be the compound with the lowest rate in WSE.

### 3.2. The Impact of WSE on the Oxidative Defense System in RIR Injury

**Figure 2:**
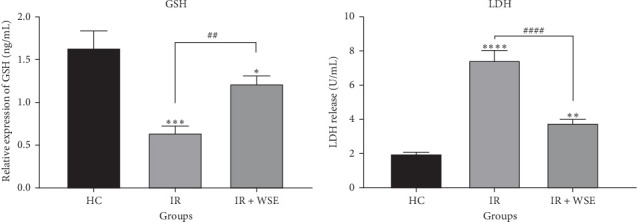
The impact of WSE, along with GSH and LDH levels, on kidney tissue following renal ischemia-reperfusion injury. The data are presented as mean ± SEM (*n* = 6). Asterisks indicate significant differences between the other groups and the HC group, while number signs indicate significant differences between the other groups and the renal IR group, as determined by Tukey's multiple range test.

As illustrated in Figure 2, rats in the renal IR group exhibited a notable decrease in GSH levels and an increase in LDH levels in their kidney tissue compared to the HC group. However, treatment with WSE led to a significant rise in GSH levels and a decrease in LDH levels in the kidney tissue of rats subjected to renal IR.

### 3.3. Gene Expression Analyses

**Figure 3:**
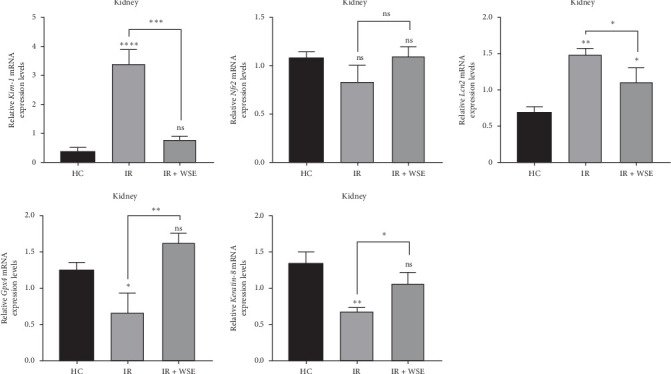
Ischemia/reperfusion-induced damage in rat kidney tissues and changes in mRNA expression of related genes as a result of pretreatment. The mRNA expression of *Kim-1*, *Nrf2*, *Lcn2*, *Gpx4*, and *Krt8*. Each bar represents the mean ± structural equation models. All experiments were repeated at least three times (*n* = 3, each group). Asterisk (*⁣*^*∗*^) indicates statistically significant difference between columns (ns *p* > 0.05, *⁣*^*∗*^*p* < 0.05, *⁣*^*∗∗*^*p* < 0.01, *⁣*^*∗∗∗*^ and *⁣*^*∗∗∗∗*^*p* < 0.001).

In this study, in order to determine the effect of WSE on mechanisms such as kidney damage, inflammation, antioxidant, detoxification, cell growth and apoptotic activities, lipid peroxidation and ferroptosis activities, and cellular differentiation, the expression levels of five different genes (*Kim-1*, *Nrf2*, *Lcn2*, *Gpx4*, and *Krt8*) known to be effective on these mechanisms were measured in all application groups. While fold changes and statistical significance values for each gene used in the study are given in Table 3, gene expression levels of the relevant genes are shown in Figure 3.

When the mRNA expression level of the *Kim-1* gene was examined, a statistically significant increase was observed in the IR group compared to the HC group (*p* < 0.0001). In the treatment group (IR + WSE), *Kim-1* mRNA levels decreased again and reached almost the HC group value. A statistically insignificant decrease in the mRNA expression level of the *Nrf2* gene was found in the IR group compared to the HC group (*p* > 0.05). In the treatment group, *Nrf2* levels were measured close to the HC group. The mRNA expression levels of the *Lcn2* gene were significantly increased in the IR group compared to the HC group (*p* < 0.01). In the treatment group, this increase was found to decrease statistically significantly and the levels approached the HC group (*p* < 0.05). A statistically significant decrease in the mRNA expression level of the *Gpx4* gene was observed in the IR group compared to the HC group (*p* < 0.05). In the treated group, *Gpx4* levels were observed to increase significantly and exceed the level of the HC group. The mRNA expression level of *Krt8* gene was statistically significantly decreased in the IR group compared to the HC group (*p* < 0.01). In the treatment group, *Krt8* levels were significantly increased but did not completely match the HC group.

## 4. Discussion

In this study, the potential protective effects of WSE were investigated in Sprague-Dawley female rats with RIR model induced kidney damage. In the study, the phytochemical content of WSE was evaluated and the effects of this extract on the expression of genes known to have effects on the abovementioned mechanisms in kidney tissue were analyzed. The findings revealed that WSE may have a protective and healing effect on kidney tissue. It was determined that the *Kim-1*, *Nrf2*, *Lcn2*, *Gpx4*, and *Krt8* genes evaluated in the study were effective on antioxidant, anti-inflammatory, iron-related cell death, and cellular differentiation processes in kidney tissue.

*Kim-1* gene expression: A decrease was observed in *Kim-1* levels, which were high in the IR group, after treatment. This situation shows that WSE can alleviate renal tubular damage. *Kim-1* expression has been identified as a powerful biomarker for detecting renal tubular damage in various kidney diseases. For example, a study has shown that *Kim-1* expression is significantly increased in AKI and this increase correlates with the severity of the disease The observed decrease in *Kim-1* levels in the treatment group may indicate protective effects of treatment approaches on the kidney. For example, peficitinib, a drug that reduces inflammation at the cellular level, has been reported to reduce *Kim-1* expression and alleviate kidney damage [21]. Similarly, the increase in *Kim-1* may be an important indicator of fibrosis and tubular inflammation in the kidney. Some studies suggest that *Kim-1* triggers inflammation in kidney tissue and that these processes play an important role in the transition to chronic kidney disease (CKD) [22]. It is emphasized in the literature that *Kim-1* expression is linked to immune responses and cellular stress and is associated with kidney fibrosis [23].

*Nrf2* gene expression: The increase in the treatment group of *Nrf2* levels, which decreased in the IR group, indicates that WSE supports cellular defense mechanisms. *Nrf2* is a key regulator of cellular antioxidant defense systems and is activated under oxidative stress conditions, inducing defense genes. The decrease in *Nrf2* expression in the IR group may indicate that oxidative stress has become uncontrolled and damage to the kidney tissue has increased. WSE is known for its high polyphenol and antioxidant content. It can be suggested that this extract may strengthen antioxidant defenses and reduce oxidative stress by activating *Nrf2*. It has been supported by many studies in the literature that polyphenols induce gene expression by increasing the nuclear translocation of *Nrf2* and thus, provide cellular protection. It has been reported in the literature that natural compounds with high polyphenol content strengthen cellular antioxidant defense mechanisms by triggering *Nrf2* activation [24]. With the activation of *Nrf2*, the expression of some genes involved in GSH synthesis increases and at the same time, defense factors such as catalase (CAT) and superoxide dismutase (SOD), which are enzymes that neutralize ROS, are activated [25]. Studies have shown that polyphenol-rich sources such as WS reduce oxidative stress markers and show protective effects by activating the *Nrf2* signaling pathway [26].

*Lcn2* gene expression: It was observed that *Lcn2* levels, which were significantly increased in the IR group, decreased in the treatment group. This finding supports the potential of WSE to suppress inflammation. Many studies have shown that *Lcn2* is an early marker of AKI and its levels increase in parallel with loss of renal function [27]. It has been reported that natural compounds can reduce *Lcn2* levels by reducing oxidative stress and inflammatory processes. For example, one study showed that polyphenol-rich extracts alleviated kidney damage and suppressed *Lcn2* expression [28].

*Gpx4* gene expression: It was observed that *Gpx4* levels, which decreased in the IR group, increased significantly in the treatment group. *Gpx4* suppresses oxidative stress by reducing lipid peroxidation and prevents ferroptosis [29]. This shows that WSE reduces oxidative stress and prevents cell death mechanisms. *Gpx4* is particularly important in suppressing oxidative stress in kidney tissue. Various studies have shown that decreased *Gpx4* levels in kidney diseases increase lipid peroxidation and lead to kidney dysfunction [30]. It has been reported in the literature that natural antioxidant sources such as WSE suppress lipid peroxidation and reduce kidney damage by increasing *Gpx4* gene expression [31].

*Krt8* gene expression: The significant increase in the treatment group of *Krt8* levels, which decreased in the IR group, shows that WSE supports cellular structure and contributes to the restructuring of kidney tissue. *Krt8* plays an important role in cellular mechanical strength and stress responses. In several studies, decreased *Krt8* expression in kidney diseases has been associated with tubular cell loss [32]. It has been reported that antioxidant treatments restore cellular integrity by supporting *Krt8* expression in kidney tissue. It has been stated that polyphenol-rich natural compounds such as WSE may have positive effects on the regulation of cytoskeletal proteins [33]. It is known that oxidative stress reduces *Krt8* expression by creating negative effects on cytoskeletal proteins. The increase in *Krt8* levels in the treatment group may be associated with the reduction of oxidative stress and the rearrangement of cellular structures [24].

Considering the findings obtained as a result of the study, it can be suggested that gene expression changes that are likely to be related to protective mechanisms in the kidneys are compatible with the physical (histological) and chemical (biochemical) signs of kidney protection. Particularly in our study, the upregulation of a gene associated with antioxidant defense and inflammation suppression, such as *Gpx4*, and a gene associated with tissue repair, such as *Krt8*, and the fact that these values were accompanied by decreased fibrosis or less damaged glomerular structure as a result of histological analysis, suggested that these upregulated genes contributed to renal protection.

WSE provides protective effects on kidney tissue through antioxidant and anti-inflammatory mechanisms, suggesting that it may be an effective treatment candidate against RIR injury. We can attribute all these positive effects of WSE to the β-sitosterol it contains. β-Sitosterol, a plant sterol commonly found in various vegetables, fruits, seeds, and nuts, has been investigated for its pharmacological effects, especially in kidney injury models [34, 35]. Studies suggest that β-sitosterol may exert protective effects against kidney injury through several possible mechanisms (anti-inflammatory effect, antioxidant activity, modulation of kidney fibrosis, regulation of lipid metabolism, cellular protection, improvement of kidney blood flow, gene expression modulation, etc.). The pharmacological effects of β-sitosterol in kidney injury models are likely multifaceted and include anti-inflammatory, antioxidant, antifibrotic, and cytoprotective mechanisms. Its ability to regulate lipid metabolism and improve kidney function makes it a potential therapeutic agent for conditions such as AKI, CKD, and diabetic nephropathy [36, 37]. In addition to its antioxidant and anti-inflammatory effects, WSE may also act on other mechanisms such as apoptosis or mitochondrial dysfunction. Natural compounds derived from plants have shown promising potential in modulating apoptosis and mitochondrial dysfunction mechanisms, which are both crucial processes in cellular health and disease. Natural compounds from plants can modulate both the intrinsic and extrinsic pathways of apoptosis, offering therapeutic potential, especially in diseases like cancer, neurodegenerative diseases, and cardiovascular disorders [38]. Mitochondria are vital for cellular energy production and their dysfunction is often associated with diseases such as Alzheimer's, Parkinson's, and ischemia/reperfusion injury. Natural plant compounds have been reported to protect mitochondria and improve their function through various mechanisms (antioxidant properties, mitochondrial biogenesis and ATP production, mitochondrial dynamics, etc.) [39]. It is possible that WSE may have such effects due to its natural compounds (especially β-sitosterol). Supporting these findings with more comprehensive and long-term studies will contribute to the full demonstration of the therapeutic potential of WSE.

## Figures and Tables

**Table 1:** Primer sequences of genes.

Gene symbol	Gene name	Forward and reverse primer sequence
*Gapdh*	Glyceraldehyde 3-phosphate dehydrogenase	F: 5'- AAACCCATCACCATCTTCCA-3' R: 5'- ATACTCAGCACCAGCATCACC-3'

*Kim-1*	Kidney injury molecule 1	F: 5'- TGTATTGTTGCCGAGTGGAG -3' R: 5'- TGTGGGTCTTGTTGGAGGA -3'

*Nrf2*	Nuclear factor erythroid 2-related factor 2	F: 5'- GCAACTCCAGAAGGAACAGG -3' R: 5'- GGAATGTCTCTGCCAAAAGC -3'

*Lcn2*	Lipocalin-2	F: 5'- TGTTCCCACCGACCAATG -3' R: 5'- GAAAGATGGAGCGGCAGA -3'

*Gpx4*	Glutathione peroxidase 4	F: 5'- TGGGAAATGCCATCAAATG -3' R: 5'- CGGCAGGTCCTTCTCTATCA -3'

*Krt8*	Keratin 8	F: 5'- ATATCCGTGTCCCGCTCTGT -3' R: 5'- TCGTTCAGGTCTTGCATGGT -3'

Abbreviations: F, forward; R, reverse.

**Table 2:** Identification of phytochemicals isolated from ethanol extract of WS.

Peak	Processing time (min)	Percentage (%)	Component name
1	21.04	0.564	Linoleic acid
2	32.523	1.067	1-Heptatriacotanol
3	33.100	21.793	Oleamid
4	34.699	0.725	Ethyl isoalcoholate
5	35.519	4.843	Hexadecanoic acid
6	35.670	4.110	Cinnamic acid
7	37.680	4.832	Brassicasterol acetate
8	38.859	62.066	β-Sitosterol

**Table 3:** Summary of fold changes and statistical significance for each gene used in the study.

Gene	Comparison	Fold change	Log2 fold change	*p*-Value
*Kim-1*	IR/HC	8.75	+3.13	0.0049 (significant)
	IR + WSE/HC	2.06	+.04	0.0225 (significant)
	IR + WSE/IR	0.24	−2.09	0.0077 (significant)

*Nrf2*	IR/HC	0.78	−0.36	0.1104 (not significant)
	IR + WSE/HC	1.02	+0.02	0.8196 (not significant
	IR + WSE/IR	1.30	+0.38	0.0926 (not significant)

*Lcn2*	IR/HC	2.16	+1.11	0.0003 (highly significant)
	IR + WSE/HC	1.59	+0.67	0.0786 (borderline)
	IR + WSE/IR	0.73	−0.44	0.0788 (borderline)

*Gpx4*	IR/HC	0.53	−0.91	0.0475 (significant)
	IR + WSE/HC	1.30	+0.38	0.0204 (significant)
	IR + WSE/IR	2.44	+1.29	0.0125 (significant)

*Krt8*	IR/HC	0.51	−0.96	0.0156 (significant)
	IR + WSE/HC	0.80	−0.33	0.0974 (not significant)
	IR + WSE/IR	1.55	+0.63	0.0392 (significant)

## Data Availability

The data that support the findings of this study are available from the corresponding author upon reasonable request.
